# Fecal bacteria and metabolite responses to dietary lysozyme in a sow model from late gestation until lactation

**DOI:** 10.1038/s41598-020-60131-1

**Published:** 2020-02-21

**Authors:** Shengyu Xu, Jiankai Shi, Yanpeng Dong, Zimei Li, Xiaoling Wu, Yan Lin, Lianqiang Che, Jian Li, Bin Feng, Zhengfeng Fang, Yong Zhuo, Jianping Wang, De Wu, Zhihua Ren, Yanping Shen

**Affiliations:** 1 Animal Nutrition Institute, Sichuan Agricultural University, Key laboratory of Animal Disease-resistant Nutrition, Ministry of Education, Key laboratory of Animal Disease-resistant Nutrition, Ministry of Agriculture and Rural Affairs, Key laboratory of Animal Disease-resistant Nutrition, Sichuan Province, Chengdu, 611130 Sichuan P. R. China; 2 College of Veterinary Medicine, Sichuan Province Key Laboratory of Animal Disease and Human Health, Key Laboratory of Environmental Hazard and Human Health of Sichuan Province, Sichuan Agricultural University, Chengdu, 611130 P. R. China; 3 Shanghai Longyou Biotechnology Co, Ltd, Shanghai, 200000 P. R. China

**Keywords:** Microbiology, Physiology, Animal physiology

## Abstract

Lysozyme (LZM) is a natural anti-bacterial protein that is found in the saliva, tears and milk of all mammals including humans. Its anti-bacterial properties result from the ability to cleave bacterial cell walls, causing bacterial death. The current study was conducted to investigate the effects of dietary LZM on fecal microbial composition and variation in metabolites in sow. The addition of LZM decreased the fecal short-chain fatty acids (SCFAs). Zonulin and endotoxin in the serum, and feces, were decreased with lysozyme supplementation. Furthermore, fecal concentrations of lipocalin-2 and the pro-inflammatory cytokine TNF-α were also decreased while the anti-inflammatory cytokine IL-10 was increased by lysozyme supplementation. 16S rRNA gene sequencing of the V3-V4 region suggested that fecal microbial levels changed at different taxonomic levels with the addition of LZM. Representative changes included the reduction of diversity between sows, decreased *Bacteroidetes*, *Actinobacteria*, *Tenericutes* and *Spirochaetes* during lactation as well as an increase in *Lactobacillus*. These findings suggest that dietary lysozyme supplementation from late gestation to lactation promote microbial changes, which would potentially be the mechanisms by which maternal metabolites and inflammatory status was altered after LZM supplementation.

## Introduction

The perinatal period is a key window for mother and offspring which brings great changes in maternal metabolism, hormones, immunity and microbiota^1,2^. Microbiota remodeling during the perinatal period may be a positive process enabling the mother to support their own health, as well as that of their offspring. Gut microbiota play a key role in the metabolism of nutrients, immune function, gut hormone secretion and provide protection from pathogens^2–5^. At the same time, microbial flora is regulated by nutrients as well as the immunological and metabolic statuses of the host. During farrowing the substantial physiological changes associated can lead to inflammatory reactions in sows^6^. Furthermore, studies have found an increase in pro-inflammatory factors during early lactation that was significantly higher than during early pregnancy or during the late stages of lactation^7,8^.

Lysozyme has been shown to be at high levels (400 mg/L) in human milk compared to the milk of other species^9^. Previous studies have found that lysozyme is beneficial to establish the bifidobacteria- and lactobacilli-rich intestinal microflora in breastfed infants, which may promote maturation of the intestinal tract^10–13^. Lysozyme is also involved in modulating the inflammatory response^14,15^. As a natural food and anti-infective substance, lysozyme has been used as an additive in the infant food and within the medical industry. In piglets it has been found that lysozyme (LZM), acting as an alternative to antibiotics, has beneficial effects on gut morphology^16,17^, immunity^18,19^, intestinal flora^19^, and growth performance^20,21^. Results of our previous study suggested that adding LZM to the diet of sows’ resulted in an increased average daily feed intake (ADFI) during lactation, shortened weaning-to-estrus interval (WEI), and improved sow and offspring health as indicated by altered inflammatory cytokines and a reduction in the piglets’ diarrhea rate^22^. However, there remains little knowledge of the maternal diversity of gut microbiota following lysozyme supplementation during the perinatal period. As a 1, 4-β-N-acetylmuramidase, lysozyme cleaves the β-1,4-glycosidic bond between the N-acetylmuramic acid and N-acetylglucosamine residues of the bacterial peptidoglycan, resulting in an incomplete cell membrane and leading to cell death^23^. This suggests that dietary lysozyme would lead to variation in the microbiota in the gut, which has been confirmed in piglets and infants^13,19,24^. Therefore, we investigated the effect on the maternal microbiota following lysozyme supplementation.

Thus, in this study we investigated the effects of adding lysozyme to maternal diets on gut permeability, gut inflammation, gut microbial and metabolite composition from the period of late gestation to lactation in a sow model.

## Results

### Fecal changes in pH and concentration of short-chain fatty acids (SCFAs)

As shown in Table 1, LZM 150 or LZM 300 had no effect on the pH and concentration of SCFAs, in sow fecal material, on day 1 of lactation. In contrast, both LZM diets increased pH values on days 7 and 21 of lactation (*P* < 0.05) when compared with the control diet. However, no difference was found between the two LZM treatments. Sows fed with the LZM 150 and LZM 300 diets showed decreased (*P* < 0.05) fecal levels of acetate, butyrate and total SCFAs on day 7 of lactation and decreased fecal levels of butyrate on day 21 of lactation when compared to sows from the control diet group. Interestingly, concentrations of total and individual SCFA increased linearly from day 1 to day 21 of lactation (Fig. S1).

**Table 1:** Effects of feeding sows diets supplemented with lysozyme on fecal SCFAs concentrations of sows.

Item	Treatment			*P-*value
	Control	LZM 150	LZM 300	
**d 1 of lactation**				
pH value	7.27 ± 0.21	7.72 ± 0.44	7.52 ± 0.29	0.08
Acetate, mg/g	6.64 ± 2.31	5.24 ± 2.00	5.33 ± 2.00	0.43
Propionate, mg/g	2.29 ± 0.89	1.67 ± 0.74	1.41 ± 0.62	0.13
Butyrate, mg/g	1.04 ± 0.54	0.58 ± 0.31	1.02 ± 0.31	0.07
Total SCFAs, mg/g	9.98 ± 3.69	7.49 ± 2.96	7.77 ± 2.57	0.31
**d 7 of lactation**				
pH value	6.93 ± 0.35^b^	7.66 ± 0.32^a^	7.37 ± 0.28^a^	<0.01
Acetate, mg/g	12.25 ± 1.12^a^	7.73 ± 0.93^b^	7.78 ± 1.90^b^	<0.01
Propionate, mg/g	3.40 ± 0.53	2.46 ± 0.21	2.54 ± 0.96	0.06
Butyrate, mg/g	1.79 ± 0.32^a^	1.02 ± 0.22^b^	1.26 ± 0.45^b^	<0.01
Total SCFAs, mg/g	17.44 ± 1.43^a^	11.21 ± 1.23^b^	11.59 ± 3.21^b^	<0.01
**d 21 of lactation**				
pH value	7.04 ± 0.33^b^	7.64 ± 0.32^a^	7.35 ± 0.48^a^	0.02
Acetate, mg/g	15.50 ± 5.52	10.82 ± 4.29	10.98 ± 3.47	0.09
Propionate, mg/g	5.26 ± 2.03	3.91 ± 2.17	3.77 ± 1.62	0.27
Butyrate, mg/g	2.69 ± 1.05^a^	1.44 ± 0.78^b^	1.46 ± 0.65^b^	0.01
Total SCFAs, mg/g	23.45 ± 8.48	16.17 ± 7.14	16.22 ± 5.61	0.09

Data are expressed as mean ± SEM. Sows were regarded as the experimental units, n = 10 for each treatment. LZM 150 = control diet + lysozyme 150 mg/kg, LZM 300 = control diet + lysozyme 300 mg/kg. SCFAs is the sum of acetate, propionate, and butyrate. a,bWithin a row, means with different superscripts are different (P < 0.05).

### Difference in gut permeability and endotoxin concentrations

**Figure 1:**
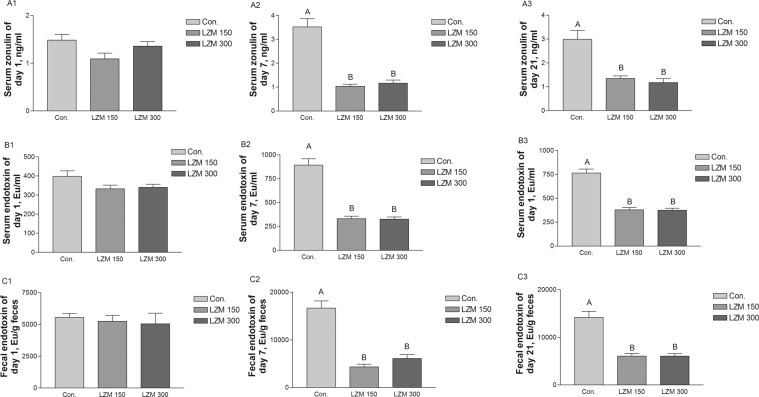
Effects of feeding sows diets supplemented with lysozyme on the serum concentrations of zonulin (A1–3) and endotoxin (B1–3) and fecal concentrations of endotoxin (C1–3). Data are presented as means ± SEM (*n* = 8). Con. = control, LZM 150 = control diet + lysozyme 150 mg/kg, LZM 300 = control diet + lysozyme 300 mg/kg. A, B, significant effect of treatment (values with different uppercase letters are significantly different, *P* < 0.01).

As a biomarker for gut permeability serum zonulin was measured. As can be seen in Fig. 1A1, no differences were found between the treatments in terms of the serum levels of zonulin on day 1 of lactation. Sows which were fed the LZM 150 and LZM 300 diets both showed decreased zonulin in their serum on days 7 and 21 of lactation (Fig. 1A2,A3, *P* < 0.05). This indicates that sows’ gut permeability was decreased by the LZM treatment. In addition, when compared with the control groups, both LZM 150 and LZM 300 treatments decreased (Fig. 1B2,B3,C2,C3, *P* < 0.05) the serum and fecal concentrations of endotoxin on days 7 and 21 of lactation, although no difference was found on day 1 of lactation (Fig. 1B1,C1).

### Inflammation state of the sows’ intestinal mucosal epithelium

**Figure 2:**
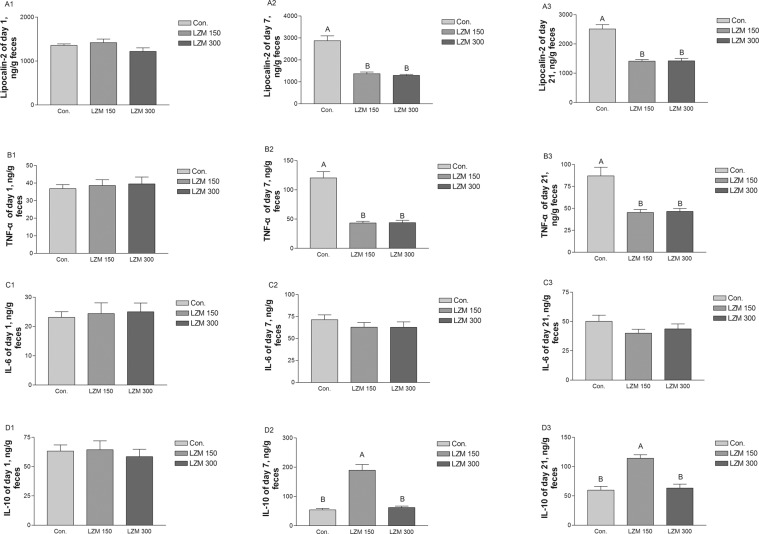
Effects of feeding sows diets supplemented with lysozyme on the serum concentrations of zonulin (A1–3) and endotoxin (B1–3) and fecal concentrations of endotoxin. Data are presented as means ± SEM (*n* = 8). TNF-α = tumor necrosis factor-α, IL-6 = interleukin-6, IL-10 = interleukin-10. Con. = control, LZM 150 = control diet + lysozyme 150 mg/kg, LZM 300 = control diet + lysozyme 300 mg/kg. A, B, significant effect of treatment (values with different uppercase letters are significantly different, *P* < 0.01).

Four biomarkers of inflammation were measured to reflect effects of LZM on the inflammatory response in sows’ intestines. As can be seen in Fig. 2, concentrations of fecal lipocalin-2 and TNF-α (pro-inflammatory cytokines) were decreased on days 7 and 21 of lactation in sows which had been fed the LZM 150 and LZM 300 diets (Fig. 2A,B). However, no significant differences were found in fecal IL-6 between the treatments (Fig. 2C1–3). In contrast, concentrations of IL-10 (anti-inflammatory cytokine) increased on days 7 and 21 of lactation for the sows on the LZM 150 diets (Fig. 2D1–3).

### Microbial diversity in sows’ feces

**Figure 3:**
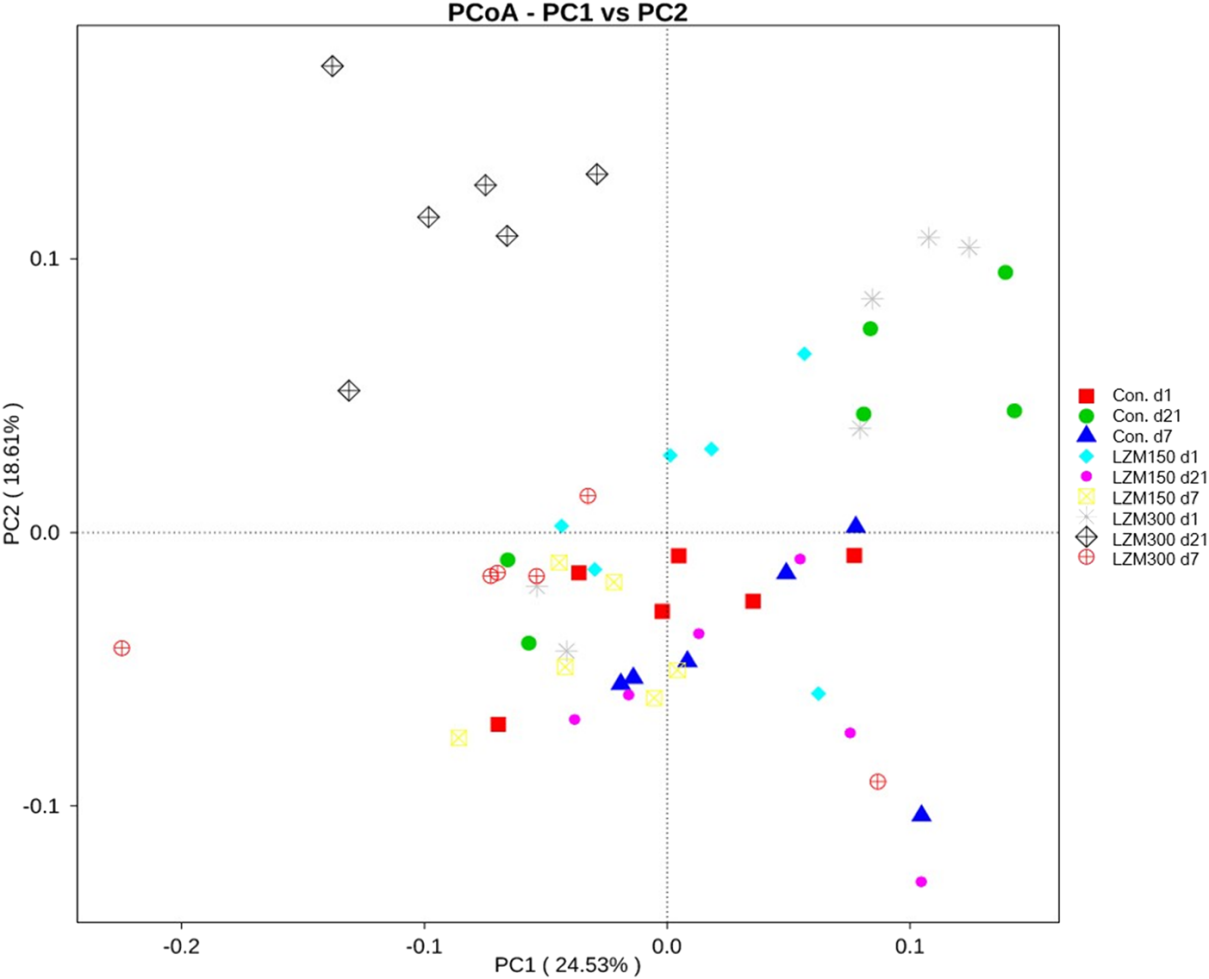
Comparison of the gut microbiota composition among treatments. Principal coordinate analysis to visualize the weighted UniFrac distances of fecal samples from individual sow. Con.d1 = control at day 1 of lactation, Con.d7 = control at day 7 of lactation, Con.d7 = control at day 21 of lactation, LZM 150 d1 = control diet + lysozyme 150 mg/kg at day 1 of lactation, LZM 150 d7 = control diet + lysozyme 150 mg/kg at day 7 of lactation, LZM 150 d21 = control diet + lysozyme 150 mg/kg at day 21 of lactation, LZM 300 d1 = control diet + lysozyme 300 mg/kg at day 1 of lactation, LZM 300 d7 = control diet + lysozyme 300 mg/kg at day 7 of lactation, LZM 300 d21 = control diet + lysozyme 300 mg/kg at day 21 of lactation.

A total of 54 fecal samples were subjected to 16S rRNA gene sequencing. Average raw reads, average effective tags and average operational taxonomic units (OTUs) for each treatment were shown in Supplementary Table S1. A set of 1,272 OTUs existed in all treatments and were thus defined as core OTUs (Fig. S2). These comprised 71.5% of the total number of OTUs, whereas 44, 37, 52, 135, 47, 105, 72, 56 and 57 OTUs were uniquely identified at Con. d1, LZM 150 d1, LZM 300 d1, Con. d7, LZM 150 d7, LZM 300 d7, Con. d21, LZM 150 d21 and LZM 300 d21, respectively (Fig. S2). To determine the bacterial diversity, the alpha and beta diversity of the fecal microbiota were assessed. We then compared the richness (observed species and Chao 1 index) and diversity (Shannon index) indices for the alpha diversity. As demonstrated in Table 2, LZM 300 diets decreased the observed species and Chao 1 index at day 21 of lactation. The lactation stage had a significant effect on fecal microbial community richness, with a Chao 1 index on day 1 of lactation being lower than at other stages (*P* < 0.01). A decrease in Shannon index (fecal microbial community diversity) with LZM supplementation at 300 mg/kg was found on day 7 and 21 of lactation (*P* < 0.01). For the analysis of beta diversity, the relationships among Control, LZM 150 and LZM 300 on day 1, 7 and 21 of lactation in the gut microbiome were examined by principal component analysis. The gut microbiota of sows showed obvious segregation in the different treatments, especially in treatment LZM300 d21 (Fig. 3) based on weighted UniFrac distance. The PERMANOVA analysis found that the bacterial community structure was significantly (*P* < 0.05) different after LZM supplementation (in Supplementary Table S2) based on the Bray-Curtis distance measures. This further indicated that lysozyme supplementation significantly affected the diversity of bacterial in the fecal matter.

**Table 2:** Effects of feeding sows diets supplemented with lysozyme on microbiota alpha diversity index of sows.

Item	time	CON	Treatment	LZM 300	*P-*value		
			LZM 150		diet	time	diet*time
Observed species	d 1	1463.33 ± 186.88	1361.50 ± 81.21	1531.67 ± 155.56^*^	0.19	0.02	<0.01
	d 7	1480.67 ± 268.49	1733.50 ± 174.18^*^	1691.67 ± 132.01^*^			
	d 21	1744.17 ± 139.69^a*^	1628.33 ± 88.08^a*^	1201.00 ± 170.23^b^			
Chao 1	d 1	1655.92 ± 202.79	1541.86 ± 121.58	1718.32 ± 166.63	0.11	<0.01	<0.01
	d 7	1672.27 ± 272.25	2072.34 ± 325.19^*^	1906.23 ± 106.26^*^			
	d 21	2034.73 ± 153.27^a*^	1909.28 ± 131.70^a*^	1359.10 ± 191.20^b^			
Shannon	d 1	7.50 ± 0.25^a^	7.32 ± 0.12^b^	7.58 ± 0.05^a*^	<0.01	<0.01	<0.001
	d 7	7.43 ± 0.21^a^	7.52 ± 0.28^a^	7.15 ± 0.53^b^			
	d 21	7.58 ± 0.12^a^	7.31 ± 0.17^ab^	6.62 ± 0.17^b^			

Data are expressed as mean ± SEM. Sows were regarded as the experimental units, n = 6 for each treatment. LZM 150 = control diet + lysozyme 150 mg/kg, LZM 300 = control diet + lysozyme 300 mg/kg. a,bWithin a row, means with different superscripts are different (P < 0.05). *Within a coloum in the same index at different day, means with asterisk denotes different (P < 0.05).

### Changes of gut microbiota composition by lysozyme in sows

The relative abundances of fecal microbiota at the phylum and genus levels for all samples during lactation are displayed in Supplementary Fig. S3. The top six dominated phyla are *Firmicutes* (73.96%), *Bacteroidetes* (11.95%), *Proteobacteria* (6.54%), *Tenericutes* (2.50%), *Euryarchaeota* (1.62%) and *Spirochaetes* (0.98%). At the genus level, *Clostridium_sensu_stricto_1* (7.74%), *Ruminococcaceae_NK4A214_group* (6.67%), *Ruminococcaceae_UCG-002* (5.72%), *[Eubacterium] _coprostanoligenes_group* (5.41%), *Ruminococcaceae_UCG-005* (4.28%), *Streptococcus* (3.74%), *Lactobacillus* (3.19%), *Escherichia-Shigella* (2.55%), *Methanobrevibacter* (1.60%) and *Bacillus* (1.30%) were the 10 dominating genera.

**Figure 4:**
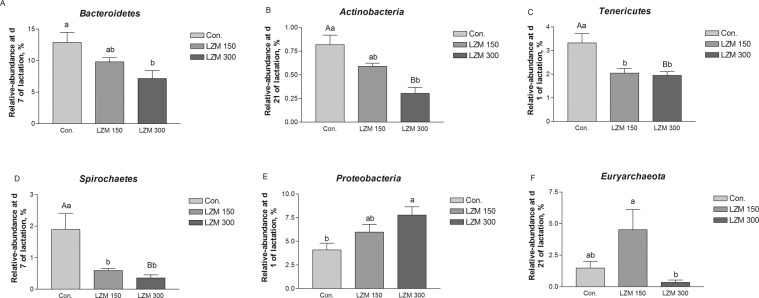
Changes in the six distinct bacterial phyla in sow gut of feeding sows diets supplemented with lysozyme. (**A**) *Bacteroidetes*, (**B**) *Actinobacteria*, (**C**) *Tenericutes*, (**D**) *Spirochaetes*, (**E**) *Proteobacteria*, and (**F**) *Euryarchaeota*. Data are presented as means ± SEM (n = 6). Con. = control, LZM 150 = control diet + lysozyme 150 mg/kg, LZM 300 = control diet + lysozyme 300 mg/kg. A, B, significant effect of treatment (values with different uppercase letters are significantly different, *P* < 0.01), and a, b significant effect of treatment (values with different lowercase letters are significantly different, *P* < 0.05).

Differences in the fecal microbiota among the three treatments at the phylum and genus levels were identified on day 1, 7 and 21 of lactation. Sows which had been fed the LZM 300 diets showed decreased relative abundance of *Bacteroidetes* and *Actinobacteria* in the gut at the phylum level on days 7 and 21 of lactation (Fig. 4A,B). In addition, sows fed LZM 150 diets showed decreased relative levels of *Tenericutes* and *Spirochaetes* in the gut on days 1 and 7 of lactation (Fig. 4C,D). However, when compared with the control group sows which had been fed the LZM 300 diets had increased relative levels of *Proteobacteria* on day 1 of lactation (Fig. 4E). The relative abundance of *Euryarchaeota* was decreased in the LZM 300 treatment group compared with the LZM 150 treatment group on day 21 of lactation (Fig. 4F). At the genus level, 23 genera relative abundances changed across the different treatments on day 1, 7 and 21 of lactation (Table 3). Sows which had been fed the LZM 150 and LZM 300 diets had decreased relative levels of *Ruminococcaceae*_UCG-005 on day 1 of lactation, decreased relative abundances of *Parabacteroides*, *Prevotella*_1, *Cellulosilyticum* and *Succinivibrio* on day 7 of lactation, and increased relative abundances of *Lactobacillus* and *Ruminococcaceae_UCG-013* on day 21 of lactation. However, sows fed the LZM 150 diets had increased relative abundance of *Methanobrevibacter* and *Ruminococcaceae*_UCG-009 on day 21 of lactation. Additionally, the LZM 300 treatment increased the relative abundances of *Romboutsia* and *Acinetobacter* on day 1 of lactation, *Ruminococcaceae*_UCG-009 on day 7 of lactation, and *Enterococcus*, *Staphylococcus* on day 21 compared with the control group. Sows which had been fed LZM 300 diets had decreased relative abundances of *Ruminococcaceae*_*UCG-010* (FDR *P* value = 0.052) on day 1 of lactation, *Ruminococcaceae_UCG-014* on day 7 of lactation, and *Terrisporobacter*, *Christensenellaceae*_R-7_group, *Oscillospira*, *Ruminococcaceae*_*UCG-010*, *Romboutsia* on day 21 of lactation.

**Table 3:** The relative abundances at genus level (%, >0.1% in at least one sample) of feeding sows diets supplemented with lysozyme.

Genera	Treatment			*P-*value	FDR
	Control	LZM 150	LZM 300		
**d 1 of lactation**					
*Ruminococcaceae_UCG-005*	5.15 ± 0.52^a^	2.97 ± 0.18^b^	3.80 ± 0.36^b^	0.008	0.015
*Ruminococcaceae_UCG-010*	1.40 ± 0.09^a^	1.26 ± 0.08^ab^	1.06 ± 0.09^b^	0.049	0.052
*Romboutsia*	0.23 ± 0.03^b^	0.23 ± 0.03^b^	0.34 ± 0.03^a^	0.031	0.040
*Acinetobacter*	0.57 ± 0.32^b^	0.35 ± 0.25^b^	2.63 ± 0.53^a^	0.005	0.019
**d 7 of lactation**					
*Parabacteroides*	0.59 ± 0.10^a^	0.27 ± 0.05^b^	0.32 ± 0.09^b^	0.033	0.040
*Prevotella_1*	0.24 ± 0.04^a^	0.12 ± 0.04^b^	0.06 ± 0.01^b^	0.007	0.016
*Ruminococcaceae_UCG-005*	4.36 ± 0.49^ab^	5.43 ± 0.54^a^	3.17 ± 0.24^b^	0.008	0.015
*Ruminococcaceae_UCG-014*	2.55 ± 0.61^a^	1.34 ± 0.20^ab^	0.86 ± 0.11^b^	0.018	0.028
*Cellulosilyticum*	0.46 ± 0.06^a^	0.29 ± 0.04^b^	0.24 ± 0.03^b^	0.005	0.023
*Ruminococcaceae_UCG-009*	0.27 ± 0.04^b^	0.32 ± 0.04^ab^	0.42 ± 0.04^a^	0.047	0.049
*Succinivibrio*	0.19 ± 0.07^a^	0.05 ± 0.01^b^	0.04 ± 0.01^b^	0.041	0.045
**d 21 of lactation**					
*Methanobrevibacter*	1.42 ± 0.52^b^	4.47 ± 1.60^a^	0.34 ± 0.18^b^	0.025	0.036
*Lactobacillus*	1.28 ± 0.45^b^	6.05 ± 1.56^a^	3.86 ± 0.56^a^	0.014	0.023
*Terrisporobacter*	3.71 ± 0.17^a^	3.32 ± 0.25^a^	2.06 ± 0.50^b^	0.008	0.016
*Enterococcus*	1.95 ± 0.58^b^	1.45 ± 0.50^b^	5.65 ± 0.41^a^	<0.001	<0.01
*Christensenellaceae_R-7_group*	2.27 ± 0.25^a^	2.56 ± 0.16^a^	1.41 ± 0.12^b^	0.006	0.016
*Staphylococcus*	0.74 ± 0.34^b^	0.91 ± 0.51^b^	2.71 ± 0.18^a^	0.004	0.012
*Oscillospira*	0.77 ± 0.09^a^	0.73 ± 0.10^a^	0.42 ± 0.09^b^	0.031	0.041
*Ruminococcaceae_UCG-009*	0.21 ± 0.05^b^	0.42 ± 0.05^a^	0.31 ± 0.04^ab^	0.014	0.024
*Ruminococcaceae_UCG-010*	1.32 ± 0.10^a^	1.03 ± 0.10^ab^	0.95 ± 0.08^b^	0.035	0.040
*Ruminococcaceae_UCG-013*	0.20 ± 0.01^b^	0.26 ± 0.03^a^	0.26 ± 0.03^a^	<0.001	<0.01
*Family_XIII_AD3011_group*	0.65 ± 0.07^ab^	0.83 ± 0.04^a^	0.40 ± 0.04^b^	<0.001	<0.01
*Romboutsia*	0.31 ± 0.02^a^	0.22 ± 0.03^ab^	0.13 ± 0.03^b^	0.007	0.020

Data are expressed as mean ± SD. Sows were regarded as the experimental units, n = 6 for each treatment. LZM 150 = control diet + lysozyme 150 mg/kg, LZM 300 = control diet + lysozyme 300 mg/kg. ^a,b^Within a row, means with different superscripts are different (*P* < 0.05).

**Figure 5:**
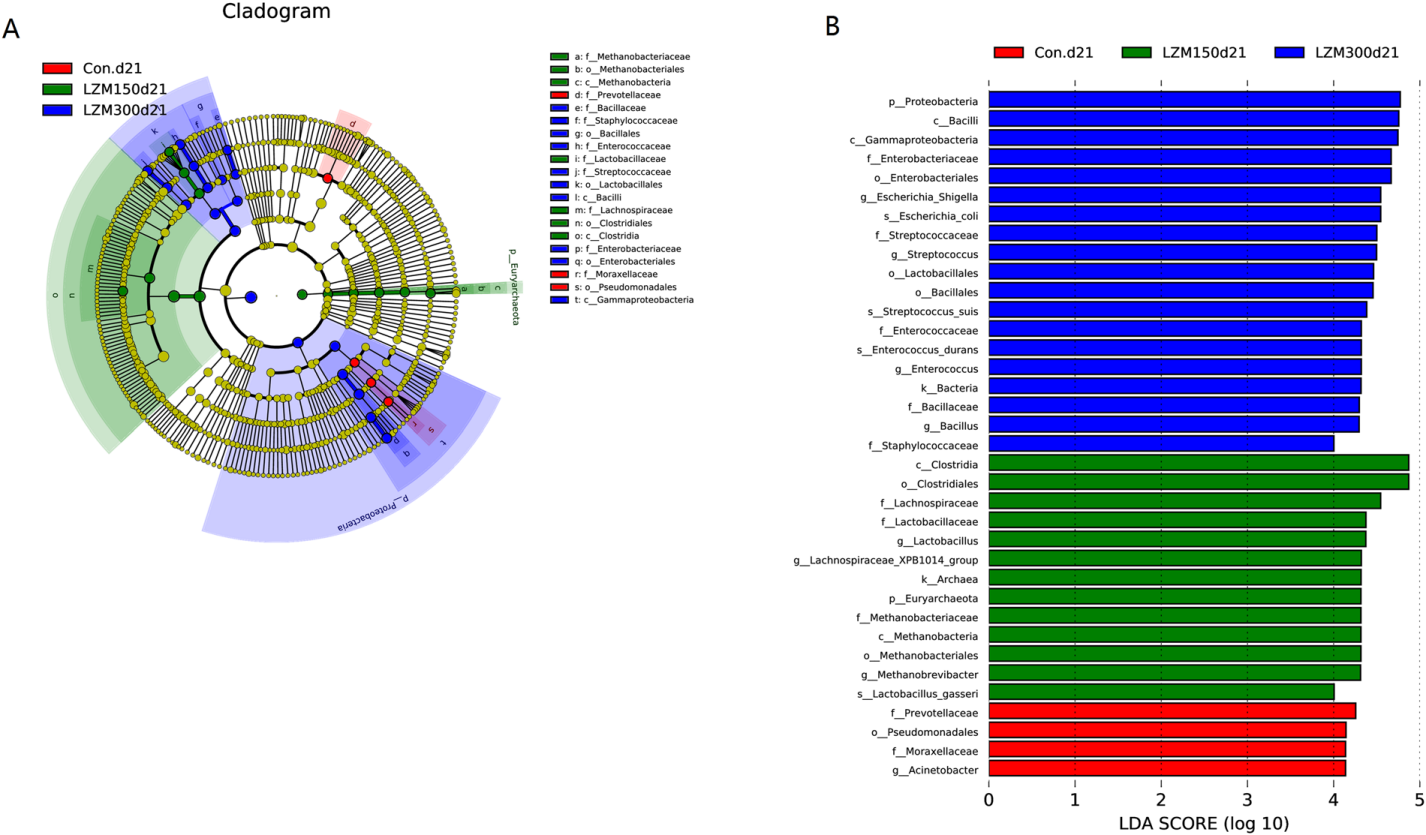
LEfSe analysis of the gut microbiota composition on d 21 of lactation of feeding sows diets supplemented with lysozyme. (**A**) Cladogram using LEfSe method indicating the phylogenetic distribution of gut microbiota in sows supplemented with lysozyme. Each successive circle represents a phylogenetic level. (**B**) Histogram of the LDA scores reveals the most differentially abundant taxa among different treatments. Con. = control, LZM 150 = control diet + lysozyme 150 mg/kg, LZM 300 = control diet + lysozyme 300 mg/kg.

Based on multiple-taxonomic LEfSe analysis of day 21 of lactation, at the phylum level *Euryarchaeota* was enriched in sows from the LZM 150 group, and *Proteobacteria* was enriched in sows from the LZM 300 group (Fig. 5A,B). At the family level, *Prevotellaceae* and *Moraxellaceae* were enriched in sows from the control group, while *Lachnospiraceae*, *Lactobacillaceae* and *Methanobacteriaceae* were enriched in sows from the LZM 150 group. *Enterobacteriaceae*, *Streptococcaceae*, *Enterococcaceae, Bacillaceae* and *Staphylococcaceae* were enriched in sows from the LZM 300 group. At the genus level, *Acinetobacter* was enriched in sows from the control group, however *Lactobacillus, Lachnospiraceae_XPB1014_group* and *Methanobacteriales* were enriched in sows from the LZM 150 group, and *Streptococcus*, *Enterococcus* and *bacillus* were enriched in sows from the LZM 300 group. In addition, LEfSe analysis at days 1 and 7 of lactation demonstrated a difference of enriched bacteria (Fig. S4). Therefore, these results demonstrate that sows’ gut microbiota composition is profoundly changed by the supplementation with LZM during lactation.

### Correlations between the gut microbiota and metabolic related parameters in sows

**Figure 6:**
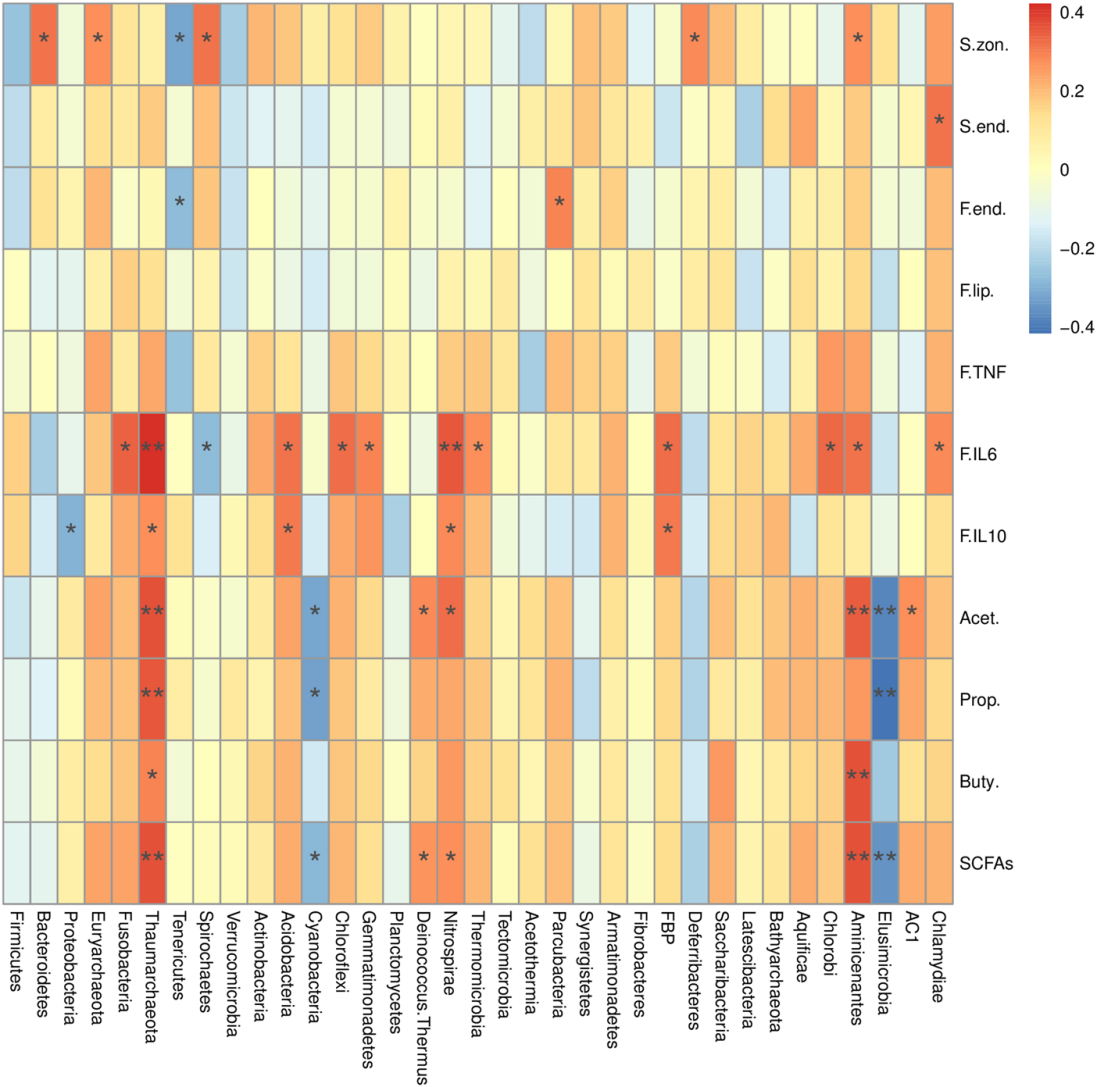
Heatmap of the spearman r correlations between the gut microbiota (phylum level) significantly modified by metabolic parameters of sows. Data are presented as means ± SEM (n = 6). *P < 0.05; **P < 0.01 (following the Spearman correlation analysis). S.zon. = serum_zonulin, S.end. = serum endotoxin, F.end = fecal endotoxin, F.lip. = fecal lipocalin-2, F.TNF = fecal TNF-α, F.IL6 = fecal IL-6, F.IL10 = fecal IL-10, Acet. = acetate, Prop. = propionate, Buty. = butyrate, SCFAs is the sum of acetate, propionate, and butyrate.

As shown in Fig. 6, at the phylum level, *Bacteroidetes*, *Euryarchaeota*, *Spirochaetes*, *Deferribacteres*, and *Aminicenantes* were positively correlated with serum zonulin (r > 0.50, *P* < 0.05). However, *Tenericutes* was negatively correlated with serum zonulin (r = −0.51, *P* < 0.01). *Fusobacteria*, *Thaumarchaeota*, *Acidobacteria*, *Chloroflexi*, *Gemmatimonadetes*, *Nitrospirae*, *Thermomicrobia*, *FBP*, *Chlorobi*, *Aminicenantes*, and *Chlamydiae* were positively correlated with fecal IL-6 (r > 0.5, *P* < 0.01). *Thaumarchaeota*, *Acidobacteria*, and *Nitrospirae* were positively correlated with fecal IL-10 (r < −0.50, *P* < 0.05). *Thaumarchaeota*, *Deinococcus.Thermus*, *Nitrospirae* and *Aminicenantes* were positively correlated with acetate, propionate, butyrate and SCFAs (r > 0.50, *P* < 0.05). *Cyanobacteria* (r = −0.52, *P* < 0.05) and *Elusimicrobia* (r = −0.59, *P* < 0.01) were negatively correlated with acetate, propionate and SCFAs. In addition, Correlations analysis at the family and genus level were shown in Fig. S5. The correlation analysis of *Bacteroidetes* with serum zonulin and LZM at various taxonomic levels was interesting. A total of 4 genera in the *Prevotellaceae* family and 2 families within the phylum *Bacteroidetes* were found to be positively correlated (r > 0.50, *P* < 0.05) with the serum zonulin, and their abundance were decreased (*P* < 0.05) with the addition of LZM (Fig. S6).

## Discussion

Lysozyme lyses a specific link in the peptidoglycan layer of bacterial cell walls resulting in cell decomposition^23^. It has been reported that piglets which consume LZM that had been highly expressed in transgenic goats’ milk showed decreased *Firmicutes* and increased *Bacteroidetes* in their feces^25^. The abundant phyla *Firmicutes* and *Bacteroidetes* accounted for 85% of microbiota in the sow feces in this study, which is in good agreement with previous studies on pigs^26,27^. It was suggested by one study that increased *Firmicutes* means the body has a higher capacity for energy acquisition from their diets^28^. However, no difference was found between the *Firmicutes* and the ratio of *Firmicutes/Bacteroidetes* in feces depending on the lysozyme treatment. The difference between these studies may be due to differing lysozyme types, dosages, and physiological stages of pigs along with differences in the duration of feeding. Zou *et al*.^24^ found that adding 100 mg/kg lysozyme (L100) to the diets of growing pig decreased cecal microflora evenness compared with control or L50 groups through 16S rRNA sequencing. In this study, we found microbial diversity decreased with lysozyme supplementation only in the LZM 300 treatment on day 21 of lactation. In addition, no difference was found in the richness of microbiota community between treatments. Interestingly, we also found that the microbiota community richness was increased with the lactation progress. This is in agreement with Cheng *et al*.^8^, who found the lowest gut microbial richness on day 3 of lactation. This may help explain how the physiological process of farrowing significantly changes the abundance of maternal gut microbiota, as well as how microflora continue to remodel and stabilize over the time.

Fermentation of dietary fiber by microbiota in the hindgut produces many SCFAs, including acetic acid, propionic acid and butyric acid^29^. With intestinal absorption, acetate is most frequently used for lipid biosynthesis^30^. Propionate tends to be a precursor of gluconeogenesis^31^. Butyrate, mainly used as an energy source, is metabolized by colonocytes and regulates the growth and death of both epithelial and immune cells^32,33^. In addition, SCFAs are signal molecules for regulating lipid and glucose metabolism by combining with free fatty acid receptors^34,35^. These contribute to the key role of SCFA in the diet-gut microbiome-host metabolism axis. Acetate, a carbohydrate fermentation product, is produced by the majority of enteric bacteria. It was more than 50% of the total SCFA in feces in this study, which is in agreement with the findings of both Zhou *et al*.^2^ and Cheng *et al*.^8^.

Additionally, we found that concentrations of total and individual SCFA increased from day 1 to day 21 of lactation, which aligns with the work of Cheng *et al*.^8^, who found an increase in butyrate from day 3 to day 14 in lactating sows. Diets supplemented with lysozyme decreased sows’ acetate, butyrate and SCFAs on day 7 of lactation, while they decreased the butyrate on day 21 of lactation compared with the control treatment. Based on our previous data, sows on lysozyme diets didn’t have any difference in back fat, weight increase during the gestation period or weight loss during the lactation period when compared with sows in the control treatment^22^. It seems that the decrease of the SCFAs didn’t affect sows’ lipid and glucose metabolism. Higher SCFAs in stool may indicate a greater amount of energy loss in feces, as suggested by Koren *et al*.^36^. The SCFAs had higher colonic absorption, with less than 5% being excreted in feces in humans^37^. When compared to production or absorption of SCFAs *per se*, fecal SCFA concentrations are more suitable for reflecting the overall net production and absorption of SCFAs in gastrointestinal tracts^38,39^. In order to assess SCFAs potential production, SCFA producing-related phylum and genera were analyzed. At the phylum level, *Bacteroides*, which mainly produces propionate, was decreased by LZM supplementation at 300 mg/kg on day 7 of lactation. The concentration of propionate had a tendency (*P* = 0.06) to decrease with LZM supplementation on that day. Despite the butyrate-producing genus *Romboutsia* being significantly decreased, *Lactobacillus* was significantly increased by the addition of LZM on day 21 of lactation. This was consistent with previous studies, which found lysozyme is beneficial to enrich *Lactobacilli* in the intestine of breastfed infants^10–12^. This possibly means that LZM supplementation in this study decreased SCFAs-producing genera and decreased the SCFAs. However, we did not find any changes in the related phenotypic indicators. This may be attributed to the reduction of SCFAs, which had not reached the threshold that causes phenotypic changes.

The increase in zonulin concentrations in blood may reflect the impaired intestinal mucosal integrity^40^. It is also the case that low intestinal microorganism richness has been associated with increased intestinal permeability in overweight pregnant women^41^. In this study, we observed that sows which had been fed LZM 150 and 300 diets had both decreased levels of zonulin in their serum on days 7 and 21 of lactation, but although no difference was found on day 1 of lactation. These findings indicate that the gut permeability of sows decreased with the addition of lysozyme suggesting lysozyme may modulate the intestinal barrier integrity in sows. While microbiota community richness was increased by the lactation progress, we didn’t observe a decrease in gut permeability in late lactation. This may due to the differences between human and sows, or could be because the microbiota richness variety hasn’t reached a threshold which induces changes in gut permeability. Correlation analysis at the phylum level found that *Bacteroidetes* and *Spirochaetes* were positively correlated with serum zonulin. The 16 s rRNA results found that LZM reduced the abundance of *Bacteroidetes* and *Spirochaetes*, meaning that serum zonulin would decrease. Although *Tenericutes* was negatively correlated with serum zonulin, and the results of 16 s rRNA found that LZM reduced the abundance of *Tenericutes*, meaning that the concentration of serum zonulin may increase, but this was not consistent with the detected results. The above results indicate that there are many influencing factors to affect serum zonulin, and the change in its concentration was a result of multiple factors. At the genus level, consistent with a zonulin concentration decrease in serum, sows which had been fed LZM 150 and LZM 300 diets had decreased *Cellulosilyticum* on day 7 of lactation, while sows which had been fed LZM 300 diets had increased *Staphylococcus* on day 21 of lactation. Spearman’s correlation found that *Cellulosilyticum* was positively correlated with serum zonulin, while *Staphylococcus* was negatively correlated with the serum zonulin (Fig. S5B). This possibly meant that sows which had been fed LZM diets had decreased phylum *Bacteroidetes* and *Spirochaetes*, genus *Cellulosilyticum* and increased *Staphylococcus*, resulting in a decrease in zonulin concentration, which in turn reflects a decline in gut permeability. Additional studies should be performed in order to demonstrate this hypothesis.

It also is reported that butyrate maintains an intestinal barrier integrity via a modulation of expression of tight junction proteins^42,43^. However, we found that sows which had been fed LZM 150 and LZM 300 diets both had a decreased concentration of butyrate and decreased endotoxin concentration in serum and feces. Endotoxin generally refers to lipopolysaccharide (lipid A) and it is a pyrogen, which is mainly present in the bacterial cell wall of gram-negative bacteria^44^. At the phylum level, we found that LZM reduced the gram-negative bacteria *Bacteroidetes*, but increased the gram-negative bacteria *Proteobacteria*. However, in terms of the proportion of microorganisms, *Bacteroidetes* was 11.95%, while *Proteobacteria* was 6.54%. This may be one important reason why LZM can reduce endotoxin levels in the serum and fecal material. In addition, phylum *Spirochaetes* decreased with the addition of LZM. One study demonstrated that many of the organisms within the *Spirochaetes* phylum cause prevalent diseases. Pathogenic members of this phylum include *Brachyspira pilosicoli* and *Brachyspira aalborgi*, which both cause intestinal spirochaetosis^45^. Based on these observations, we suggest that while the addition of LZM does not increase the content of butyrate, it reduces intestinal permeability and endotoxin content as well as reducing level of *Spirochaetes*, which may be beneficial to intestinal health.

At the phylum level, we observed that sows which had been fed LZM 150 and LZM 300 diets decreased *Actinobacteria* and *Tenericutes* on days 1 and 21 of lactation. The two kinds of phylum were thought to be associated with inflammation responses. Patients with inflammatory bowel disease^46^ as well as obese Ossabaw minipigs^47^ have high levels of *Actinobacteria* at the colon. In general, chronic or low-grade inflammation is a hallmark of obesity and metabolic disorders^48,49^. It has also been found that, along with gestational weight gain induced by the addition of fat, the phylum *Actinobacteria* increased. From the inflammatory indicators they found that the sows were in high-grade inflammation^2^. Similarly, high levels of phylum *Tenericutes* have been found in diet-induced obese mice^50^ and obese Göttingen pigs^47^. We found that *Tenericutes* decreased with the addition of LZM. This effect of LZM was similar with the report of Everard *et al*.^51^ who found that the addition of probiotic yeast decreased the abundance of *Tenericutes*. Given these observations, we hypothesized that sows fed LZM diets present with low-grade inflammation compared with control. Consistent with this hypothesis, lipocalin-2 and pro-inflammatory cytokines TNF-α in feces were decreased in the LZM 150 and 300 groups, while the IL-10 (anti-inflammatory cytokines) increased in the LZM 150 group on days 7 and 21 of lactation. Consistent with these findings, our previous study found that LZM 150 and LZM 300 increased IL-10 in serum on day 7 of sow lactation, and had a tendency to enhance IL-10 in serum on day 1 of lactation^22^, and previous studies also found that lysozyme are involved in modulating the inflammatory response^14,15^. Cytokines regulate both immune responses^52^ and nutrient distribution which, characterized by proinflammatory cytokines, induce more nutrients to enter the immune response rather than being used for growth^53,54^. Lower TNF-α was found in pigs which had been fed lysozyme diets than in control groups^20^. Phylum *Proteobacteria* contains multiple pathogens and also has proinflammatory properties^55^. High levels of *Proteobacteria* are a potential diagnostic feature of epithelial dysfunction and dysbiosis in gut microbiota^56,57^. We found that LZM supplementation at 300 mg/kg enriched the *Proteobacteria* on day 1 of lactation. However, we didn’t find any cytokine differences between treatments. Based on these findings, we propose that pro-inflammatory or anti-inflammatory factors are affected by changes in microbial flora; not only by a single microbe, but by the results of multiple microbial interactions. However, the possible mechanisms for how gut microbiota interact to cause intestinal inflammation in perinatal period maternal body require further study.

In conclusion, this study suggested that gut microbiota and their metabolites were altered in sows following the addition of LZM to their diets during the period of late gestation to lactation. Typical changes involve the expansion of diversity among treatments, an increase in *Lactobacillus* as well as a decrease in *Romboutsia*, *Spirochaetes*, *Actinobacteria* and *Tenericutes* in LZM 300 treatment. Microbial changes triggered by the additional of LZM during late gestation to lactation could potentially be the mechanism for the positive effects seen of LZM on gut permeability and gut health regulation, as well as maternal anti-inflammation. Until now, there has been little knowledge about the maternal microbial alterations induced by LZM supplementation, meaning that special attention should be given to this area in future research. Using lysozyme in maternal diets from late gestation to lactation may be a beneficial way of preventing gut permeability, related inflammation status and metabolites disturbances with a remodeled microbial ecosystem.

## Materials and Methods

All animal procedures in this study were approved by the Animal Care and Use committee of Sichuan Agricultural University, under ethical approval number DKY-S20156137. All the experiments were performed in accordance with the guidelines and regulations of the Animal Care and Ethical Committee of the Sichuan Agricultural University.

### Animals and experimental design

A total of 60 Yorkshire × Landrace sows (3–6 parity, 14.07 ± 2.58 mm backfat thickness) were used in this study, which was designed as a single factorial arrangement of treatments including the control group (basal diet, *n* = 20), LZM 150 diet group (basal diet + 150 mg/kg lysozyme, concentration in accordance with the results of antibacterial tests, data not shown, *n* = 20), and the LZM 300 diet group (basal diet + 300 mg/kg lysozyme, *n* = 20). The sows received treatment diets from day 85 of gestation (usually farrowing at day 114 of gestation) to the end of weaning (day 21 of lactation). Based on a corn-soybean meal, the diet was formulated to meet nutritional requirements recommended by the National Research Council 2012 (NRC 2012). The gestation basal diet included 3.04 Mcal digestible energy (DE)/kg, 14.65% of crude protein, 0.69% Lys, 0.85% calcium, and 0.67% phosphorus, while the lactation basal diet included 3.29 Mcal DE/kg, 17.54% of crude protein, 0.99% Lys, 0.99% calcium and 0.68% phosphorus. None of the diets included antibiotics, probiotics or other medicines. The LZM used in the study was given by Shanghai Longyou Biotechnology Co., Ltd. Enzyme activity is 5000U/mg.

During gestation (day 85 to 106), all sows were housed in individual gestation stalls. On day 107 of pregnancy, sows were transferred to individual farrowing crates. Before farrowing, sows were fed an average of 3.5 kg/d diet. On farrowing days, sows were fed 0.5 kg of feed, which was gradually increased by 1.0 kg/d until the maximum feeding amount was reached as described as before^22^. Sows were then allowed to feed *ad libitum* throughout the rest of their lactation days. Sows had free access to water in this study.

### Sample collection

On day 1 of lactation, 10 sows were randomly selected from each treatment. Used the same sows to collect samples at days 7 and 21 of lactation. Fresh feces were collected by massaging the rectum of each sow^8^. Sows had no diarrhea or other disease before sampling. On day 1, samples were collected within two hours after farrowing. Fresh feces were stored in three sterile tubes and kept in liquid nitrogen before being transferred to −80 °C. The 5 mL blood sample was obtained from the same 10 sows’ ear veins for each treatment group before morning meal on days 1, 7 and 21 of lactation. Serum samples were obtained by centrifuging blood for 10 min at 3,000 g at 4 °C, and were then stored at −20 °C for further analysis^22^.

### Analysis of fecal pH value and SCFAs

Fecal pH values (n = 10) were detected following the method of Topping *et al*.^58^. Briefly, distilled water was used to dilute 0.5 g of feces at a ratio of 1:2 (weight/volume) by a blender. Fecal homogenate was then centrifuged (20 °C) for 15 min at 3,000 g, after which the supernatant was measured by pH meter^58^ (PHS-3C pH, Shanghai, China).

Concentrations of the fecal SCFAs (acetate, propionate and butyrate, n = 10) were analyzed as described by Chen *et al*.^59^. A 2 g feces sample was suspended in distilled water (5 mL) and allowed to stand for 30 min, then it was centrifuged for 10 min at 12,000 g at 4 °C. The 2 mL of sample supernatant was then transferred and mixed with 0.4 mL of metaphosphoric acid. The sample placed at 4 °C for 30 min, and centrifuged again for 10 min at 12,000 g at 4 °C before being transferred to 1.2 mL of supernatant and mixed with 15.2 μL of crotonic acid (210 mmol/L, internal standard), after which 0.3 mL liquid was mixed with 0.3 mL methanol^59^. Finally, 1 μL of supernatant was used to analyze of SCFAs by a gas chromatograph^59^ (GC, Varian CP-3800 GC, USA; capillary column 30 m × 0.32 mm × 0.25 μm film thickness). The minimal detection limit for each SCFA was 0.1 mmol/L.

### Analysis of metabolic biomarker

The gut health related metabolic biomarkers lipocalin-2, endotoxin, interleukin-6 (IL-6), tumor necrosis factor-α (TNF-α), interleukin-10 (IL-10) of fecal as well as zonulin and endotoxin of serum (n = 8, 8 samples were randomly selected from the 10 sows of which were randomly selected for the sample collection of each treatment at day 1 of lactation. Samples came from the same 8 sows of days 7 and 21 of lactation of each treatment.) were measured using porcine enzyme-linked immunosorbent assay (ELISA) Kits (R&D Systems Inc., Minneapolis, MN, USA). The biomarker analysis was performed according to the manufacturer’s instructions.

### Analysis of bacterial community

The Mo Bio PowerFecal^TM^ DNA Isolation Kit (MO BIO Laboratories, Carlsbad, CA, USA) was used to extract the microbial DNA of thawed fecal sample (0.5 g, n = 6, 6 samples were randomly selected from the 8 sows of which were randomly selected for the metabolic biomarker analysis of each treatment at day 1 of lactation. Samples came from the same 6 sows of days 7 and 21 of lactation of each treatment.). The nucleic acid/protein analyzer (Beckman DU-800, Beckman Coulter, Inc., CA, USA) was utilized to examine the concentration and purity of DNA. Then the DNA samples were sent to perform amplicon pyrosequencing on Illumina HiSeq PE250 platforms and bioinformatics analyses at Novogene Bioinformatics Technology in Beijing, China. The V4 hypervariable region of the 16S rRNA gene was amplified using a forward primer 515 f (5′-GTGCCAGCMGCCGCGGTAA-3′) and a reverse primer 806r (5′-GGACTACHVGGGTWTCTAAT-3′) as described as before^2^.

The linker sequence, barcode sequence in the reads, homopolymer runs exceeding 6 bp, primer mismatches, and sequence lengths shorter than 100 bp were removed. Paired-end reads from the original DNA fragments were merged using FLASH version 1.2.7^60^. Then Quantitative Insights Into Microbial Ecology^61^ (QIIME) version 1.7.0. was applied to filter out sequences containing ambiguous bases (N) or low-quality bases of the merging data. Following removed the chimeric sequences in the splicing sequence. High-quality tags were clustered into OTUs utilizing Uparse v7.0.1001 (http://drive5.com/uparse/) at 97% sequence similarity. The Ribosomal Database Project (RDP) classifier Version 2.2 (http://github.com/rdpstaff/) was applied to assign taxonomy for 16S rRNA gene sequences. The relative abundance of each OTU was examined at different taxonomic levels. A Venn diagram was generated for comparison among the OTUs of the three treatments. Alpha diversity values for each sample were assessed by Qiime 1.7.0. To identify bacterial taxa differentially represented among different treatments at genus or higher taxonomy levels, the linear discriminant analysis coupled with effect size (LEfSe) was applied^62^.

### Statistical analysis

The original data were checked using Grubbs’ test method. If |Xp − ^−^X | å λ (α, n) S, the Xp was considered as the outlier. Before using parametric analyses, descriptive statistics were performed to check the normality and homogeneity of variances. Data of relative abundance at the phylum and genus level in fecal matter were log-transformed before statistical analysis. The data were analyzed using the General Linear Model (GLM) procedures of SAS (V9.3, SAS Institute Inc., Cary, NC, USA) followed by a DUNCAN analysis for multiple comparison when the F test in the analysis of variance table was significant. The statistical model was: *Y*_*ij*_ = *μ* + *t*_*i*_ + *e*_*ij*_ where *Yij* is the analyzed variable, *μ* is the overall mean, *t* is the effect of treatment (_*i*_ = 1, 2, 3), and *e* is the residual error (_*i*_ = 1, 2, 3, _*j*_ = 1…10 or 6). Normality of residual check was performed using PROC UNIVARIATE with Normal and Plot options in SAS. Data for the figures (Figs. 1,2,4, S1) were analyzed using a one-way ANOVA followed by Tukey’s test for multiple comparisons using GraphPad Prism analysis software. All data are shown as mean ± SEM. Differences of *p* < 0.05 were considered significant when, whereas *p* < 0.10 was considered a trend.

To analyze correlations between microbiota and metabolic parameters, Spearman’s correlation in R 3.0.2 with the Rstudio 0.97.310 package and gplots package for the heat map were used. Differences of *p* < 0.05 were considered significant. Data were corrected by false discovery rate analysis according to the Benjamini–Hochberg method with an α of <0.05^63^.

## Supplementary information

Supplementary material.
