# Supplementary material for: Fecal bacteria and metabolite responses to dietary lysozyme in a sow model from late gestation until lactation
Source: Sci Rep. 2020 Feb 21;10:3210. doi: 10.1038/s41598-020-60131-1 (PMC7035255; doi:10.1038/s41598-020-60131-1)
Supplement: Supplementary file 1 — Supplementary material. [file 41598_2020_60131_MOESM1_ESM.pdf]

Supplementary Material

Fecal bacteria and metabolite responses to dietary lysozyme in a sow model from late gestation until lactation

Shengyu Xu[+,*], Jiankai Shi[+], Yanpeng Dong, Zimei Li, Xiaoling Wu, Yan Lin, Lianqiang Che, Jian Li, Bin Feng, Zhengfeng Fang, Zhuo Yong, Jianping Wang, De Wu, Zhihua Ren and Yanping Shen

[+] These authors contributed equally to the work.

*Correspondence and requests for materials should be addressed to S.X. (email: shengyu_x@hotmail.com)

Table S1. Average raw reads, effective tags and OTUs of fecal microbial community from d 1 to d 21 of lactation

	Average raw reads	Average effective tags	Average OTUs
D 1 of lactation			
Control	93907	87274	1691
LZM 150	87652	82109	1563
LZM 300	90011	84109	1763
D 7 of lactation			
Control	92703	87277	1676
LZM 150	87743	78176	1972
LZM 300	91428	83020	1980
D 21 of lactation			
Control	90382	83196	2068
LZM 150	94420	87309	1927
LZM 300	90072	80487	1368

LZM 150 = control diet + lysozyme 150 mg/kg, LZM 300 = control diet + lysozyme 300 mg/kg.

Table S2. PERMANOVA test of the differences in bacterial community structure based on weighted UniFrac distance measures

	Con. d1	LZM 150 d1	LZM 300 d1	Con. d7	LZM 150 d7	LZM 300 d7	Con. d21	LZM 150 d21
LZM 300 d21	**0.004**	**0.002**	**0.002**	**0.004**	**0.005**	**0.005**	**0.003**	**0.003**
LZM 150 d21	**0.003**	**0.008**					**0.025**	
Con. d21	**0.013**	0.076						
LZM 300 d7	**0.002**	0.072					**0.043**	0.07
LZM 150 d7	0.074	**0.003**		**0.011**		0.156	0.051	**0.027**
Con. d7	0.076	0.167				**0.025**	**0.037**	0.261
LZM 300 d1	0.063	0.094		**0.036**	**0.028**	**0.024**	0.297	**0.008**
LZM 150 d1	0.140							

Values in bold means significant difference between two groups at $P < 0.05$. Con. d1 = control diet on day 1 of lactation, LZM 150 d1 = control diet + lysozyme 150 mg/kg on day 1 of lactation, LZM 300 d1 = control diet + lysozyme 300 mg/kg on day 1 of lactation, Con. d7 = control diet on day 7 of lactation, LZM 150 d7 = control diet + lysozyme 150 mg/kg on day 7 of lactation, LZM 300 d7 = control diet + lysozyme 300 mg/kg on day 7 of lactation, Con. d21 = control diet on day 21 of lactation, LZM 150 d21 = control diet + lysozyme 150 mg/kg on day 21 of lactation, LZM 300 d21 = control diet + lysozyme 300 mg/kg on day 21 of lactation.

A1

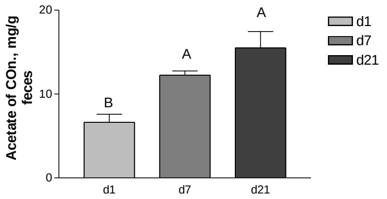

A2

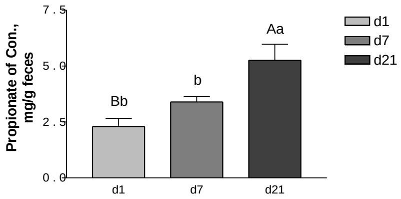

A3

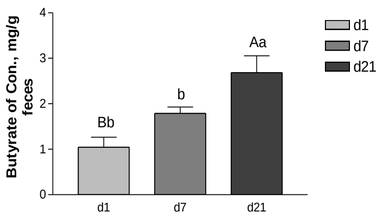

A4

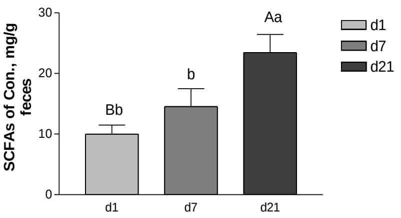

B1

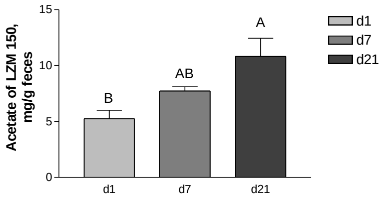

B2

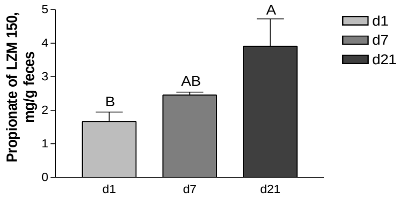

B3

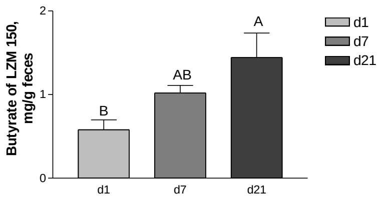

B4

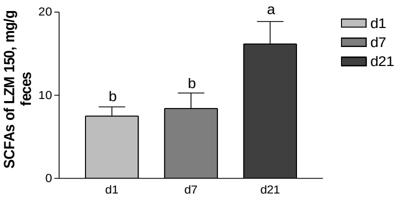

C1

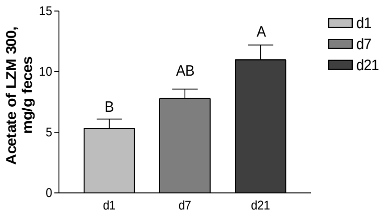

C2

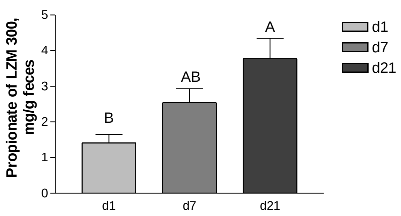

C3

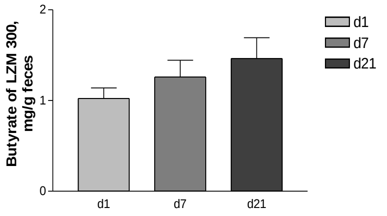

C4

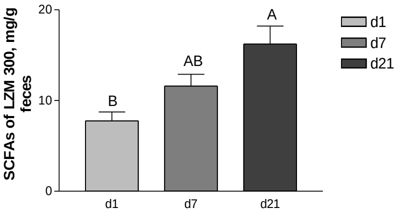

Figure S1. Fecal concentrations of intestinal microbial metabolites in sows from d 1 to d 21 of

lactation. (A1) acetate of control group, (A2) propionate of control group, (A3) butyrate of control group, (A4) SCFAs of control group, (B1) acetate of LZM 150 group, (B2) propionate of LZM 150 group, (B3) butyrate of LZM 150 group, (B4) SCFAs of LZM 150 group, (C1) acetate of LZM 300 group, (C2) propionate of LZM 300 group, (C3) butyrate of LZM 300 group, and (C4) SCFAs of LZM 300 group. Data are presented as means ± SEM (n = 10). d1, day 1 of lactation; d7, day 7 of lactation; d21, day 21 of lactation. SCFAs, short-chain fatty acid; SCFAs is the sum of acetate, propionate, and butyrate. Values with different lowercase letters are significantly different, $P < 0.05$; values with different uppercase letters are significantly different, $P < 0.01$.

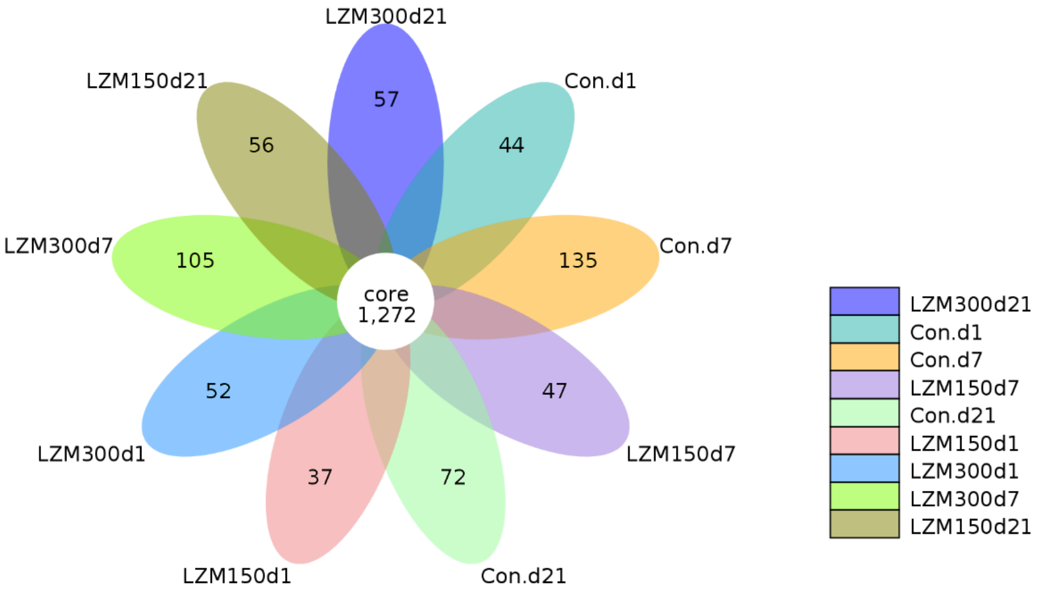

Figure S2. Venn diagrams were generated to compare OTUs between the different treatments at different stages. Venn diagram was generated to describe the common and unique OTUs among treatments at different day of lactation. Con.d1 = control at day 1 of lactation, Con.d7 = control at day 7 of lactation, Con.d21 = control at day 21 of lactation, LZM 150 d1 = control diet + lysozyme 150 mg/kg at day 1 of lactation, LZM 150 d7 = control diet + lysozyme 150 mg/kg at day 7 of lactation, LZM 150 d21 = control diet + lysozyme 150 mg/kg at day 21 of lactation, LZM 300 d1 = control diet + lysozyme 300 mg/kg at day 1 of lactation, LZM 300 d7 = control diet + lysozyme 300 mg/kg at day 7 of lactation, LZM 300 d21 = control diet + lysozyme 300 mg/kg at day 21 of lactation.

A

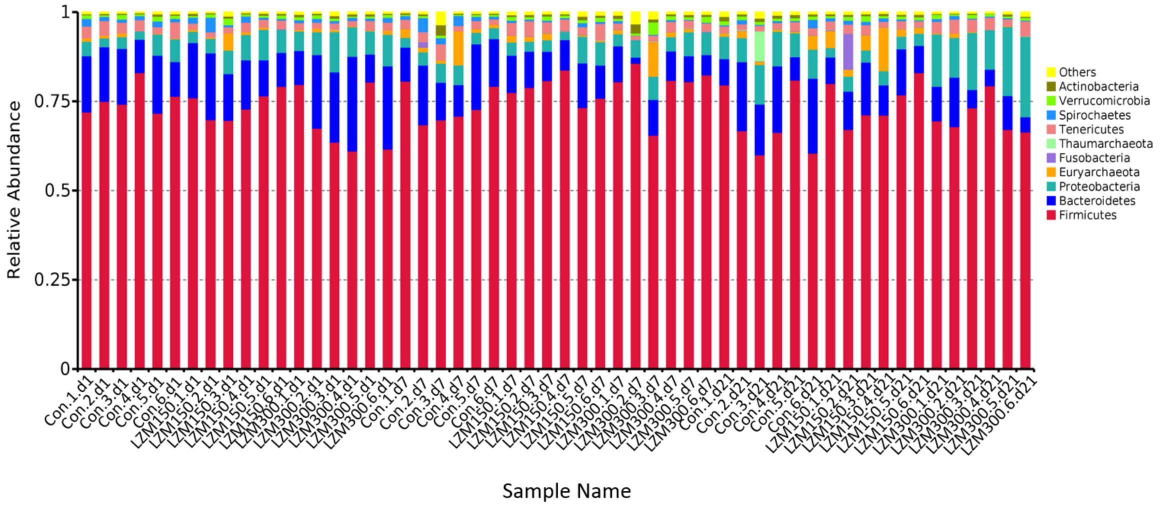

B

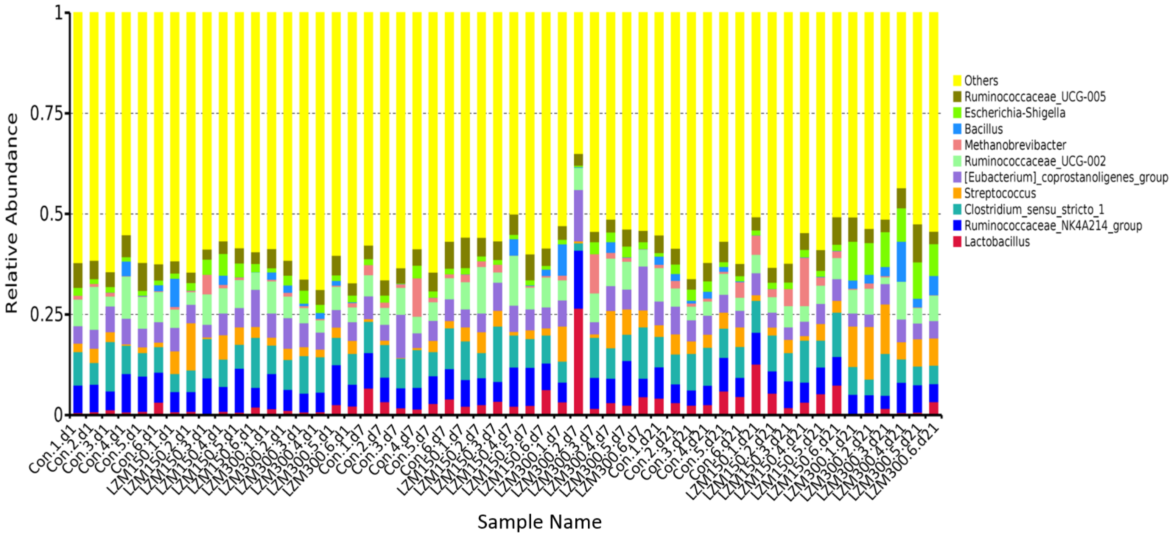

Figure S3. The relative abundance at phylum level and genera level at each stage of lactation.

(A) The relative abundance at phylum level. (B) The relative abundance at genera level.

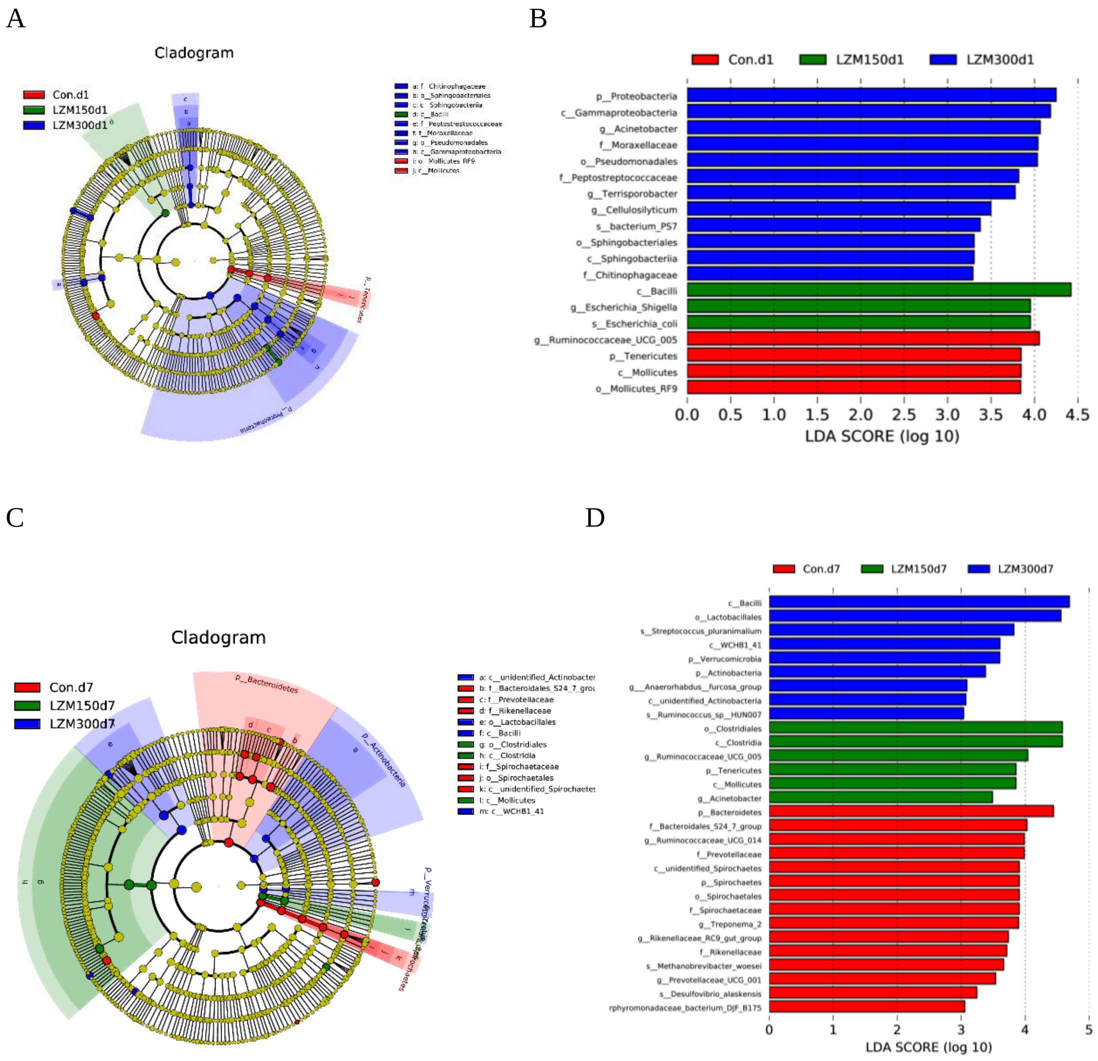

Figure S4. LEfSe analysis of the gut microbiota composition on d 1 (A, B) and 7 (C, D) of lactation of feeding sows diets supplemented with lysozyme. (A, C) Cladogram using LEfSe method indicating the phylogenetic distribution of gut microbiota in sows supplemented with lysozyme on d 1 and 7 of lactation. Each successive circle represents a phylogenetic level. (B, D) Histogram of the LDA scores reveals the most differentially abundant taxa among different treatments on d 1 and 7 of lactation. Con. = control, LZM 150 = control diet + lysozyme 150 mg/kg, LZM 300 = control diet + lysozyme 300 mg/kg.

A

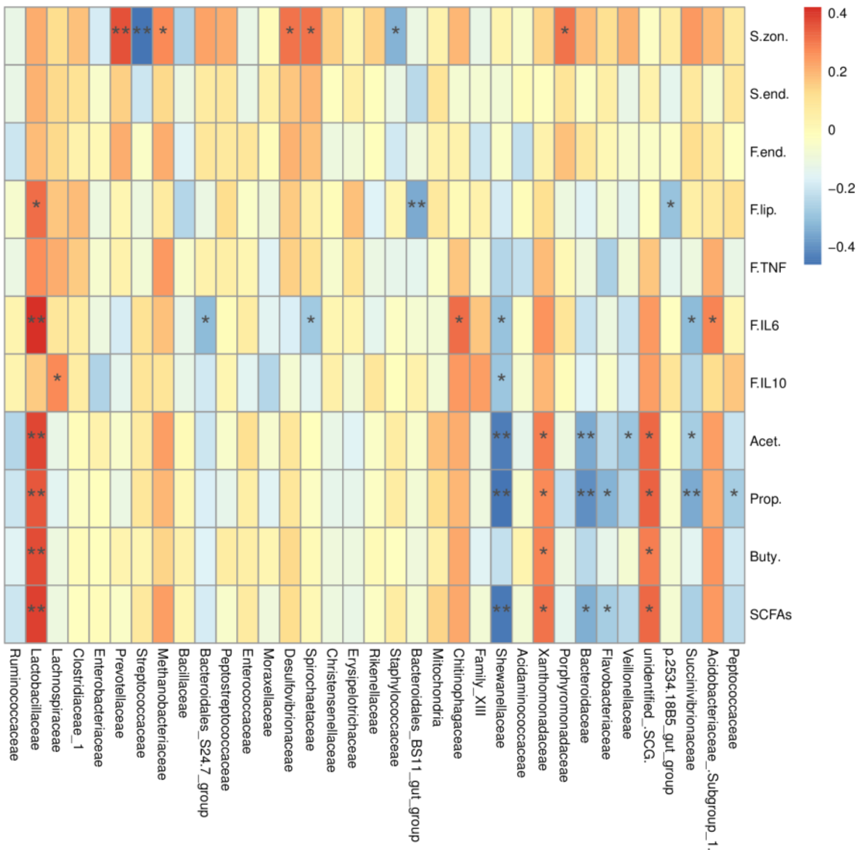
0.4
0.2
0
−0.2
−0.4
S.zon.
S.end.
F.end.
F.lip.
F.TNF
F.IL6
F.IL10
Acet.
Prop.
Buty.
SCFAs
Ruminococcaceae
Lactobacillaceae
Lachnospiraceae
Clostridiaceae_1
Enterobacteriaceae
Prevotellaceae
Streptococcaceae
Methanobacteriaceae
Bacillaceae
Bacteroidales_S24.7_group
Peptostreptococcaceae
Enterococcaceae
Moraxellaceae
Desulfovibrionaceae
Spirochaetaceae
Christensenellaceae
Erysipelotrichaceae
Rikenellaceae
Staphylococcaceae
Bacteroidales_BS11_gut_group
Mitochondria
Chitinophagaceae
Family_XIII
Shewanellaceae
Acidaminococcaceae
Xanthomonadaceae
Porphyromonadaceae
Bacteroidaceae
Flavobacteriaceae
Veillonellaceae
unidentified_SCG.
p.2534.18B5_gut_group
Succinivibrionaceae
Acidobacteriaceae_.Subgroup_1.
Peptococcaceae

B

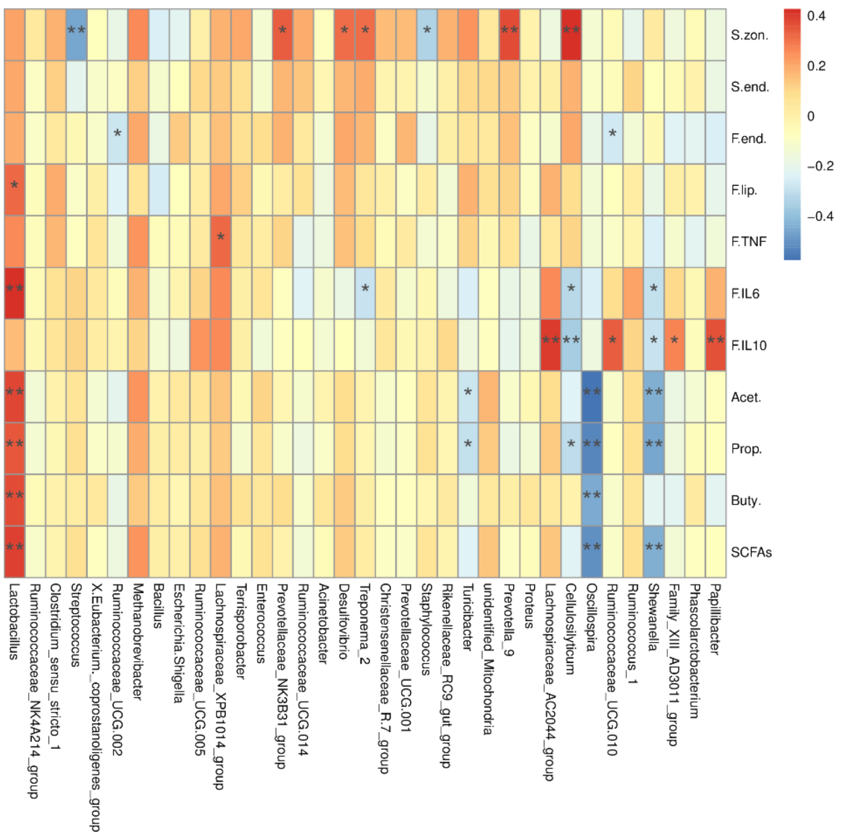
0.4
0.2
0
−0.2
−0.4
S.zon.
S.end.
F.end.
F.lip.
F.TNF
F.IL6
F.IL10
Acet.
Prop.
Buty.
SCFAs
Lactobacillus
Ruminococcaceae_NK4A214_group
Clostridium_sensu_stricto_1
Streptococcus
X.Eubacterium._coprostanoligenes_group
Ruminococcaceae_UCG.002
Methanobrevibacter
Bacillus
Escherichia.Shigella
Ruminococcaceae_UCG.005
Lachnospiraceae_XPB1014_group
Terrisporobacter
Enterococcus
Prevotellaceae_NK3B31_group
Ruminococcaceae_UCG.014
Acinetobacter
Desulfovibrio
Treponema_2
Christensenellaceae_R.7_group
Prevotellaceae_UCG.001
Staphylococcus
Rikenellaceae_RC9_gut_group
Turicibacter
unidentified_Mitochondria
Prevotella_9
Proteus
Lachnospiraceae_AC2044_group
Cellulosilyticum
Oscillospira
Ruminococcaceae_UCG.010
Ruminococcus_1
Shewanella
Family_XIII_AD3011_group
Phascolarctobacterium
Papillibacter

Figure S5. Heatmap of the spearman r correlations between the gut microbiota (A. family level, B. genera level) and metabolic parameters of sows. Data are presented as means ± SEM (n = 6). *$P < 0.05$; **$P < 0.01$ (following the Spearman correlation analysis). S.zon. = serum_zonulin, S.end. = serum endotoxin, F.end = fecal endotoxin, F.lip. = fecal lipocalin-2, F.TNF = fecal TNF-α, F.IL6 = fecal IL-6, F.IL10 = fecal IL-10, Acet. = acetate, Prop. = propionate, Buty. = butyrate, SCFAs is the sum of acetate, propionate, and butyrate.

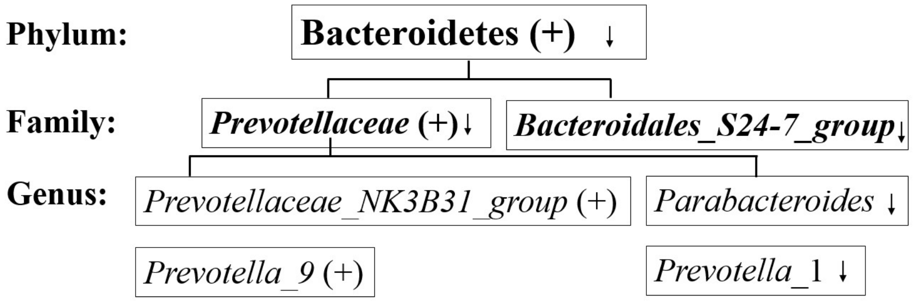

Figure S6. Relationship of *Bacteroidetes* with serum zonulin and LZM at various taxonomic levels. The "+" sign indicates a positive correlation (r > 0.5, $P < 0.05$) with the serum Zonulin, and the "↓" sign indicates that the addition of LZM reduced ($P < 0.05$) the abundance of the bacteria.
